# Diversity and Localization of Bacterial Endosymbionts from Whitefly Species Collected in Brazil

**DOI:** 10.1371/journal.pone.0108363

**Published:** 2014-09-26

**Authors:** Julio Massaharu Marubayashi, Adi Kliot, Valdir Atsushi Yuki, Jorge Alberto Marques Rezende, Renate Krause-Sakate, Marcelo Agenor Pavan, Murad Ghanim

**Affiliations:** 1 Department of Entomology, Agricultural Research Organization, The Volcani Center, Bet Dagan, Israel; 2 Departamento de Fitossanidade, Faculdade de Ciencias Agronomicas, UNESP, Botucatu, Sao Paolo, Brazil; 3 Instituto Agronomico de Campinas, Campinas, Sao Paolo, Brazil; 4 Departamento de Fitopatologia e Nematologia, Escola Superior de Agricultura, Piracicaba, Sao Paolo, Brazil; International Atomic Energy Agency, Austria

## Abstract

Whiteflies (Hemiptera: Aleyrodidae) are sap-sucking insect pests, and some cause serious damage in agricultural crops by direct feeding and by transmitting plant viruses. Whiteflies maintain close associations with bacterial endosymbionts that can significantly influence their biology. All whitefly species harbor a primary endosymbiont, and a diverse array of secondary endosymbionts. In this study, we surveyed 34 whitefly populations collected from the states of Sao Paulo, Bahia, Minas Gerais and Parana in Brazil, for species identification and for infection with secondary endosymbionts. Sequencing the mitochondrial Cytochrome Oxidase I gene revealed the existence of five whitefly species: The sweetpotato whitefly *Bemisia tabaci* B biotype (recently termed Middle East-Asia Minor 1 or MEAM1), the greenhouse whitefly *Trialeurodes vaporariorum*, *B. tabaci* A biotype (recently termed New World 2 or NW2) collected only from Euphorbia, the Acacia whitefly *Tetraleurodes acaciae* and *Bemisia tuberculata* both were detected only on cassava. Sequencing rRNA genes showed that *Hamiltonella* and *Rickettsia* were highly prevalent in all MEAM1 populations, while *Cardinium* was close to fixation in only three populations. Surprisingly, some MEAM1 individuals and one NW2 population were infected with *Fritschea. Arsenopnohus* was the only endosymbiont detected in *T. vaporariorum.* In *T. acaciae* and *B. tuberculata* populations collected from cassava, *Wolbachia* was fixed in *B. tuberculata* and was highly prevalent in *T. acaciae*. Interestingly, while *B. tuberculata* was additionally infected with *Arsenophonus, T. acaciae* was infected with *Cardinium* and *Fritschea.* Fluorescence *in situ* hybridization analysis on representative individuals showed that *Hamiltonella*, *Arsenopnohus* and *Fritschea* were localized inside the bacteriome, *Cardinium* and *Wolbachia* exhibited dual localization patterns inside and outside the bacteriome, and *Rickettsia* showed strict localization outside the bacteriome. This study is the first survey of whitely populations collected in Brazil, and provides further insights into the complexity of infection with secondary endosymionts in whiteflies.

## Introduction

The whitefly, *Bemisia tabaci* Gennadius (Hemiptera: Aleyrodidae) is one of the most devastating insect pests of the 20th century, being cosmopolitan and polyphagous [1]. In addition to the direct damage caused by feeding on the plant phloem and secretion of honeydew, *B. tabaci* is known as vector of more than 100 plant viruses from different genera including *Begomovirus, Crinivirus, Carlavirus* and others [2], [3].

*B. tabaci* is considered a complex of cryptic species, which are also sometimes termed “biotypes”, and are characterized as genetic variants or haplotypes that differ in molecular and biological characteristics [4]. Based on the molecular and biological aspects of *B. tabaci* it is classified into about 35 species [1], [5], [6], [7]. For example, biotypes B and B2 belong to the new termed Middle East-Asia Minor 1 (MEAM1) species; the biotypes Q, J, L, Sub-Saharan Africa, the Silver-leafing biotypes and Mediterranean all belong to the group termed Africa/Middle East/Asia Minor, and the biotypes known as A, C, D, F, Jatropha, N, R and Sida belong to the New World group that includes New World (NW) and New World 2 (NW2) species [1], [5]. Based on the new classification, the species MEAM1, NW and NW2 were recently investigated in Brazil [8]. In addition to *B. tabaci,* other whitefly species such as *Bemisia tuberculata*, *Tetraleurodes acaciae* and *Trialeurodes vaporariorum* are known to be present in Brazil. For example *B. tuberculata* has been shown to colonize cassava plants in the states of São Paulo, Mato Grosso and Rio de Janeiro [9], [10], [11], while the greenhouse whitefly *T. vaporariorum*, was associated with outbreaks in 2003 in horticultural and ornamental crops in the state of São Paulo [12].

Whiteflies, similar to many other arthropods, are usually infected with bacterial endosymbionts that may influence their biology and their interaction with other organisms or the environment [13]. Whiteflies harbor the primary obligatory endosymbiont *Portiera aleyrodidarum* [14], [15], which is essential for the species persistence and for complementing the insect’s unbalanced diet that consists mainly sugars from the plant phloem. Besides *Portiera,* whitefly populations from around the world were reported to harbor a diverse array of facultative secondary endosymbionts which are not essential for the species persistence and those include the Gammaproteobacteria *Arsenophonus* (Enterobacteriales), *Hamiltonella* (Enterobacteriales) [16], [17], *Fritschea* (Chlamydiales) [18], *Cardinium* (Bacteroidetes) [19], the alphaproteobacteria *Rickettsia* (Rickettsiales) [20], *Wolbachia* (Rickettsiales) [21], [22], and *Orientia-like organism* [23]. Several whitefly populations around the world were surveyed for infection with endosymbionts and a clear association between biotypes and secondary symbionts were often observed. For example, in populations tested from Israel *Hamiltonella* was detected only in the B biotype, *Wolbachia* and *Arsenophonus* only in the Q biotype, and *Rickettsia* in both biotypes [24]. None of the populations from Israel were infected with *Fritschea* or *Cardinium. Fritschea* has only been detected in the A biotype (NW2) from the United States [25], and only *Arsenophonus* has been associated with *T. vaporariorum* [16]. *Cardinium* was detected in B and Q biotype populations from China [26], Q populations from Croatia [27], [28], and France [29]. Additional *T. vaporariorum* populations were analyzed from Croatia and were reported to be infected with *Portiera* and *Hamiltonell*a in addition to the previously reported infection with *Arsenophonus* [27]. Other whitefly species such as the ash whitefly *Siphoninus phillyreae* (Haliday) from Croatia were surveyed and the same endosymbionts were detected similar to other whitefly species [28].

In the current study, different populations representing five whitefly species collected in four states in Brazil were surveyed for infection with secondary endosymbionts, and the identified endosymbionts were localized in adult individuals using fluorescence *in situ* hybridization (FISH) analysis. The results provide new infections with endosymbionts in whitefly species that have not been reported before, adding further complexity to the already known endosymbiont bacterial infections among whitefly populations around the world.

## Results

### Identification of whitefly species

Altogether 34 populations were analyzed in this study. The populations were collected from four states in Brazil between 2010–2012, from different locations and host plants as shown in Table 1. Varying numbers of individuals, depending on availability, from each population were analyzed for infection with six different secondary endosymbionts: *Hamiltonella, Rickettsia, Cardinium, Wolbachia, Arsenophonus* and *Fritschea*. PCR targeting *Portiera,* the primary endosymbionts, was performed as a positive control for the reaction. PCR and sequencing analyses were further performed for determining the whitefly species and biotypes using the COI gene amplification and sequencing and microsatellite primers as detailed in the methods section. Twenty two populations were identified as *B. tabaci* MEAM1 species (B biotype), while one population was identified as the NW2 (Table 1). Other whitefly species that were identified based on COI gene amplification are: four populations of *B. tuberculata*, three populations of *T. acaciae* and four populations of *T. vaporariorum* (Table 1).

**Table 1:** Whitefly species collected and tested in this study.

Population	Whitefly species	Collection location	Host plant
1	MEAM1^1^	Votuporanga	Couve
2	MEAM1	São José do Rio Preto	Melon
3	MEAM1	Lins	Pepper
4	MEAM1	Campinas	Soybean
5	MEAM1	Estiva Gerbi	Tomato
6	MEAM1	Jaboticabal	Tomato
7	MEAM1	Registro	Jilo
8	MEAM1	Presidente Prudente	Watermelon
9	MEAM1	Ourinhos	Soybean
10	MEAM1	Assis	Soybean
11	MEAM1	Marília	Watermelon
12	MEAM1	Piracicaba	Tomato
13	MEAM1	Guapiara	Cauliflower
14	MEAM1	Itapeva	Soybean
15	MEAM1	Capão Bonito	Potato
16	MEAM1	Itararé	Potato
17	MEAM1	Patos de Minas - (MG)^3^	Tomato
18	MEAM1	Dom Basílio - (BA)^4^	PassionFruit
19	MEAM1	Londrina (PR)^5^	Bean
20	MEAM1	Londrina (PR)	Cotton
21	MEAM1	Bastos	Euphorbia
22	MEAM1	Botucatu	Eggplant
23	NW2^2^	Bastos	Euphorbia
24	*T. vaporariorum*	Apiaí	Green Bean
25	*T. vaporariorum*	Bragança Paulista	Tomato
26	*T. vaporariorum*	Bom Sucesso do Itararé	Cucumber
27	*T. vaporariorum*	Botucatu	Eggplant
28	*B. tuberculata*	Votuporanga	Cassava
29	*B. tuberculata*	Cardoso	Cassava
30	*B. tuberculata*	Assis	Cassava
31	*B. tuberculata*	Candido Mota	Cassava
32	*T. acaciae*	Lins 1	Cassava
33	*T. acaciae*	Lins 2	Cassava
34	*T. acaciae*	Itararé	Cassava

All collections were made in the state of São Paulo unless another indicated in parenthesis near the collection location.

1 MEAM1: *Bemisia tabaci* Middle East Asia Minor 1; NW2^2^: *Bemisia tabaci* New World 2; ^3^MG: Minas Gerais; ^4^BA: Bahia; ^5^PR: Parana.

### *B. tabaci* infection with secondary endosymbionts

**Figure 1:**
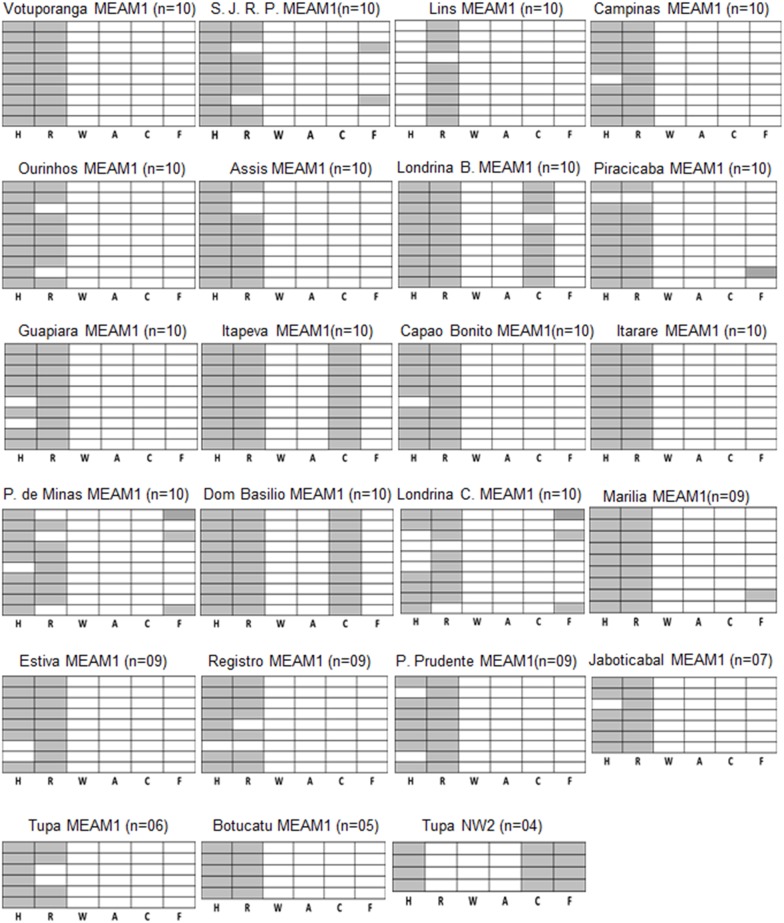
Individual and mixed infection by secondary symbionts in *B.tabaci* populations collected in this study. Twenty two MEAM1 (B biotype) and one NW2 (A biotype) populations were collected and surveyed. Each box represents one population. Vertical columns represent the different symbionts tested as indicated in the base of each column, and each horizontal column represents one individual that was tested for the presence of the six different symbionts. Shaded boxes represent positive infection with the tested symbiont. The geographical origin of the populations, the species and the number of individuals tested are indicated at the top of each box (see Table 1 for full names).

Individual whiteflies were analyzed for infection with the six secondary endosymbionts using genus-specific primers (Table 2), and each individual was tested for infection with *Portiera*, the primary endosymbionts, as a positive control. Figure 1 shows the infection with endosymbionts of all individuals that were tested from each population and the mixed infections with multiple endosymbionts within each individual. For example ten individuals from the population collected in Votuporanga from cabbage were tested, and all these individuals were co-infected with *Hamiltonella* and *Rickettsia*, while the other secondary endosymbionts were not detected. The most prevalent co-infection observed in the tested populations was individuals doubly infected with *Hamiltonella* and *Rickettsia* (64.20%). Most of the individuals from three populations collected in Itapeva, Dom Basilio and Londrina were simultaneously infected with *Hamiltonella, Rickettsia* and *Cardinium*, and the prevalence of triply infected individuals was 14.22%. Other single, double and triple infections from *B. tabaci* MEAM1 populations were also observed and those included individuals infected with only *Rickettsia* or only *Hamiltonella,* individuals infected with *Hamiltonella* and *Fritschea* (Patos de Minas) and two individuals infected with *Hamiltonella, Rickettsia* and *Fritschea*. Interestingly, only three individuals among total of 204 individuals that were analyzed were not infected with any secondary endosymbionts while the rest were infected with at least one endosymbiont (98.04% infection rate). 91.67% of the individuals were infected with *Rickettsia*, 87.25% with *Hamiltonella,* 14.22% with *Cardinium*, 4.90% with *Fritschea*. The four individuals in the only population that was identified as NW2 were infected with *Hamiltonella, Cardinium* and *Fritschea*.

**Table 2:** List of primers used for identification of endosymbionts.

Gene	Primer sequence (5′>3′)	Annealing (°C)/product size (bp)	Reference
*Portiera* 16 SrRNA	F - CGCCCGCCGCGCCCCGCGCCCGTCCCGCCGCCCCCGCCCG R- CCGTCAATTCMTTTGAGTTT	60/550	[69]
*Rickettsia* 16 SrRNA	F - GCTCAGAACGAACGCTATC R -GAAGGAAAGCATCTCTGC	60/900	[20]
*Hamiltonella*16 S rRNA	F - TGAGTAAAGTCTGGAATCTGG R -AGTTCAAGACCGCAACCTC	60/700	[21]
*Wolbachia* 16 SrRNA	F - CGGGGGAAAAATTTATTGCT R -AGCTGTAATACAGAAAGTAAA	55/700	[70]
*Arsenophonus*23 S rRNA	F - CGTTTGATGAATTCATAGTCAAA R -GGTCCTCCAGTTAGTGTTACCCAAC	60/600	[14]
*Cardinium* 16 SrRNA	F - GCGGTGTAAAATGAGCGTG R -ACCTMTTCTTAACTCAAGCCT	58/400	[19]
*Fritschea* 23 SrRNA	F - GATGCCTTGGCATTGATAGGCGATGAAGGAR - TGGCTCATCATGCAAAAGGCA	60/600	[18]

### *T. vaporariorum, T. acacia* and *B. tuberculata* infection with secondary endosymbionts

**Figure 2:**
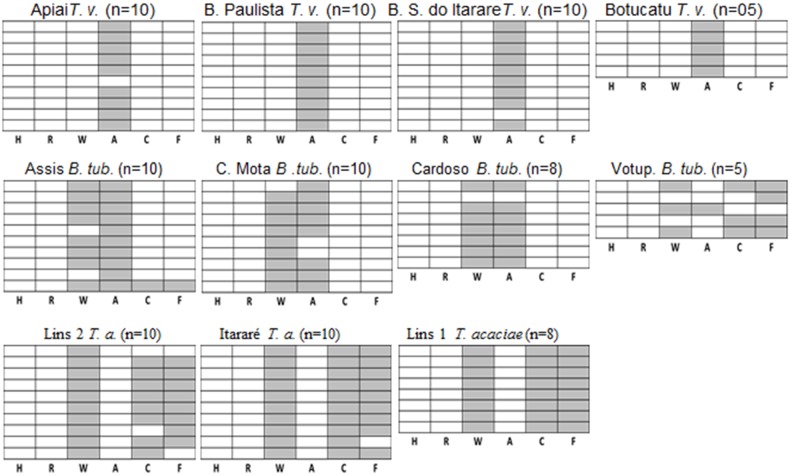
Individual and mixed infections with secondary endosymbionts in additional whitefly species analyzed in this study. Four populations of *T. vaporariorum (T. v.)*, 4 populations of *B. tuberculata* (*B. tub.)* and 3 populations of *T. acaciae* (*T. a.)* were collected and analyzed in this study (See legend to figure 2 for more details).

The prevalence of *T. vaporariorum* was much lower than *B. tabaci* in the surveyed regions. Thirty five *T. vaporariurom* individuals from four different regions of the State of Sao Paulo were tested for infection with the six different endosymbionts (Fig. 2). The primary endosymbiont *Portiera* was detected in all individuals, while among the secondary endosymbionts only *Arsenophonus* was detected as a single infection (94.28% of the tested individuals) in all individuals except one individual from Apiai and a second individual from Bom sucesso do itarare, both were uninfected with none of the secondary endosymbionts (Fig. 2). Two additional whitefly species were detected in the survey we performed. Both species, *B. tuberculata* and *T. acaciae*, were detected only on cassava plants (Table 1). Four *B. tuberculata* and three *T. acacia* populations were analyzed for infections with endosymbionts.

Among *B. tuberculata* tested individuals, 96.97% were infected with at least one endosymbiont, while only one individual was not infected with any secondary endosymbiont (Fig. 2). *Wolbachia* and *Arsenophonus* were detected in single or mixed infections in all four populations analyzed. *Arsenophonus* was the most prevalent endosymbiont with 81.82% infection rate and *Wolbachia* with 84.85% infection rate. Interestingly, *Fritschea* appeared in 15.15% infection rate, while *Cardinium* with 12.12%, and both appeared in single or mixed infections. One individual was infected with *Fritschea,* one individual with only *Fritschea* and *Cardinium* both collected in Votuporanga, and one individual collected from Assis was infected with the four endosymbionts detected in *B. tuberculata*: *Wolbachia, Arsenophonus*, *Fritschea* and *Cardinium*.

Among *T. acaciae* populations, three endosymbionts were detected: *Wolbachia, Cardinium* and *Fritschea.* All tested individuals were infected with at least one endosymbiont (Fig. 2). *Wolbachia* appears to be fixed in *T. acaciae* populations with all individuals harboring this endosymbiont. The triple infection with the three detected endosymbionts was the most prevalent infection pattern with 85.71% of the individuals showing this infection pattern. Among the 28 tested individuals only one individual showed single infection with *Wolbachia,* one individual showed double infection with *Wolbachia* and *Cardinium* and one individual showed double infection with *Wolbachia* and *Fritschea* (Fig. 2).

### Localization of secondary endosymbionts in whitefly species collected in Brazil

All detected secondary endosymbionts in the populations collected in Brazil were localized in all developmental stages using FISH analysis. All the previously reported localization patterns for endosymbionts in whiteflies from complete confinement within the bacteriome to complete localization outside the bacteriome, and dual localization patterns inside and outside the bacteriome were observed.

**Figure 3:**
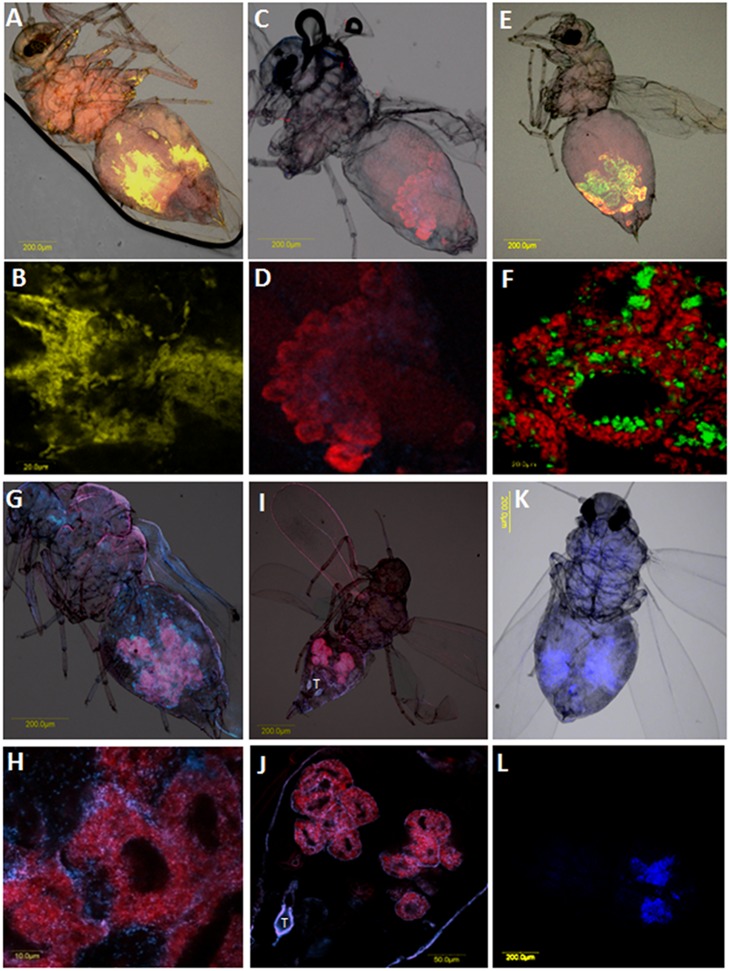
FISH of selected specimens collected in this study for specific localization of secondary endosymbionts. A–B: FISH of *Arsenophonus* (yellow) and *Portiera* (red) in *T. vaporariorum* under bright field (A) and dark field (B). C–D: FISH of *Fritschea* (blue) and *Portiera* (red) in NW2 under bright field (C) and dark field (D). E–F: FISH of *Hamiltonella* (green) and *Portiera* (red) in MEAM1 under bright field (E) and dark field (F). G–H: FISH of *Rickettsia* (blue) and *Portiera* (red) in MEAM1 under bright field (G) and dark field (H). I–J: FISH of *Cardinium* (blue) and *Portiera* (red) in *T. acaciae* under bright field (I) and dark field (J). K–L: FISH of *Wolbachia* (blue) in *B. tuberculata* under bright field (K) and dark field (L). T: testicle.

Four endosymbionts were detected in *B. tabaci: Hamiltonella, Rickettsia, Cardinium* and *Fritschea. Hamiltonella* was detected only in *B. tabaci* MEAM1 and NW2 species (Fig. 1) and localization experiments showed that this bacterium is always confined in the bacteriosome cells, co-localizing with *Portiera,* the primary endosymbionts (Fig. 3). *Hamiltonella* cells were localized is small patches inside bacteriocyte cells and did not occupy the whole cavity of the cell. This localization pattern was observed in all developmental stages: eggs, nymphal stages and adults. *Rickettsia* was localized outside the bacteriome and occupied most of the body cavity, but not bacteriocyte cells. This localization pattern was observed in all developmental stages. Close inspection of *Rickettsia* localization patterns showed that it infects many organs.

Two additional endosymbionts that were localized inside the bacteriome are *Arsenophonus* and *Fritschea. Arsenophonus*, which was not detected from *B. tabaci* and *T. acaciae,* but was present in *T. vaporariorum* and *B. tuberculate*, localized in both species inside bacteriocyte cells with *Portiera,* the primary endosymbiont. *Arsenophonus* localized in patches and appeared in rod-shaped structures, unlike the other endosymbionts. *Fritschea* was localized in all tested whitefly species except in *T. vaporariorum* also inside bacteriocute cells, however, the localization signal was weak and the concentration of the bacterium seemed very low.

Two endosymbionts that were detected in this study, *Wolbachia* and *Cardinium*, exhibited dual localization pattern inside and outside the bacteriome (Fig. 3). These two bacteria were detected in *B. tabaci, B. tuberculata* and *T. acaciae.* The localization pattern in the three species did not differ for the two endosymbionts; however both symbionts appeared in very low concentration. As in previous reports, *Wolbachia* is localized on the circumference or within the bacteriocytes with the primary endosymbiont. Additionally, although *Wolbachia* can be found outside bacteriocyte cells, it is always localized adjacent to those cells in the abdomen and not seen in other parts of the insect such as the thorax and the head.

## Discussion

This study presents, for the first time, a comprehensive survey of whitefly species that were detected in four states in eastern Brazil: São Paulo, Parana, Bahia and Minas Gerais. Altogether 34 populations were collected from different host plants and tested for whitefly species identity and infections with bacterial endosymbionts using PCR and sequencing (Tables 1 & 2). The majority of *B. tabaci* populations were collected in São Paulo while only few populations were collected in the remaining states. Twenty three of the collected populations in the four states were identified as MEAM1 species, four were *T. vaporariorum,* four *B. tuberculate,* three populations were identified as *T. acaciae* and one population as NW2. All other whitefly species that were not MEAM1 were collected only in São Paulo state. MEAM1 populations were collected from different host plants, suggesting that none of the host plants was a preferred host for this species, which emphasizes its known polyphagy.

*B. tabaci* populations collected in this study all belonged to the MEAM1 species and only one population collected from Euphorbia was identified as NW2 (Table 1). MEAM1 populations were found to harbor *Hamiltonella, Rickettsia, Cardinium* and surprisingly some individuals were also infected with *Fritschea*, which also infected all individuals from the only NW2 population. The identity of *Fritschea* was verified by sequencing the amplified 23 S rRNA gene fragments. Previous surveys of whitefly species in some parts of the world that included MEAM1, MED and others, such as in Israel [24], China [26], Croatia [27], [28] and some European and African countries [29], revealed that *Fritschea* does not infect any of the surveyed populations, and that this bacterium was only reported from the USA infecting the A biotype (NW) [18]. To assess the variability of the bacterial species infecting the surveyed populations tested in this study, the 16 S or 23 S rRNA gene sequences were obtained. The results revealed that for each bacterial species no variability was observed and it is likely that one strain from each bacterium was detected in the surveyed populations in this study, indicating that single infections, or single introductions of whiteflies infected with those endosymbionts are the origin of the observed bacterial populations. For example, sequences from *Fritschea* that was detected in MEAM1 and NW2 showed 100% homology, suggesting that the same strain of *Fritschea* colonize both whitefly species, which may indicate a horizontal transfer or ancestral single infection with *Fritschea* between the two species by sharing host plant [34], [35]. Endosymbionts can be injected into the plant vascular system from which they can be acquired and established in new arthropod hosts [36]. A recent study has shown that salivary glands can indeed be infected by endosymbionts, as in the case of *Cardinium* in *Scaphoideus titanus* [37], [38]. An additional study has shown that *Rickettsia* in whiteflies can be horizontally transmitted through the plant vascular system [39], presenting its ability to colonize distantly related insect species through common plant hosts.

*T. vaporariorum* populations collected from São Paulo were found to be infected only with *Arsenophonus,* unlike *T. vaporariorum* populations previously tested in Croatia that were shown to be infected with *Hamiltonella* and *Arsenophonus* [27], [28]. The only other whitefly species that was found to be infected with *Arsenophonus* in this study is *B. tuberculata,* which was found to colonize only cassava plants. It is hard to hypothesize how this symbiont infects these distantly related insects and again it might be a recent horizontal transfer or an infection of an ancient ancestor. Other symbiont strains that were shared between distantly related whitefly species collected in this study are *Cardinium* which was shared between MEAM1, NW2, *B. tuberculata* and *T. acaciae,* and *Fritschea* which was also shared between all whitefly species except for *T. vaporariorum.* Other examples included *B. tuberculata* infection with *Wolbachia, Cardinium* and *Fritschea,* infection of *B. tabaci* MEAM1 with *Hamiltonella, Rickettsia, Cardinium and Fritschea,* the infection of NW2 with *Hamiltonella, Cardinium and Fritschea* and the infection of *T. acaciae* with *Wolbachia, Cardinium* and *Fritschea.* Some populations showed very low infection rates with some of the symbionts such as the infection of some MEAM1 populations with *Fritschea* and *Cardinium.* This result can be attributed to recent introduction of new whitefly populations with specific symbiont infections into Brazil, or instability or rarity of some whitefly-endosymbiont combinations under certain conditions such as climate, predation, agricultural practices and more. Several internal factors may influence maximum infection rates in the same individuals. These factors include competition for space and resources among two or more endosymbionts [33], [40], or on the contrary, positive interaction, cooperation and dependence of one endosymbiont on the other [41]. Another important factor is the insect host response to the presence of these symbionts which in most cases will influence the bacterial community residing within this host. In general, infection with secondary symbionts, without reaching fixation suggests that those bacteria are not essential for the insect, while bacteria that reached fixation or close to fixation indicate mutualistic relationships. For example, *Hamiltonella* was shown to infect only 40% of the pea aphid individuals tested from California, while only 0–40% were infected with *Rickettsia* [42], [43], [44], [45], [46], [47]. *Hamiltonella* reached fixation in *B. tabaci* populations, like those tested from Israel [24], [27], [28], [29], indicating some mutualistic or obligatory relationships, with the insect or the primary endosymbiont as *Hamiltonella* was always localized inside bacteriome cells with the primary endosmbiont *Portiera.* Such interactions might be complementing the host’s diet, increasing its fitness or coping with environmental conditions such as resistance to extreme conditions.

*B. tuberculata* and *T. acaciae* were not previously surveyed with regard to infection with secondary endosymbionts. Both species strictly share the same host plant and can be found in sympatry on cassava, and were not detected on other plant hosts. Although it is not known when those two species became specialized on cassava in Brazil, it is not surprising that both species share some secondary endosymbionts including *Wolbachia, Cardinium* and *Fritschea.* Interestingly, *Fritschea* was previously reported only from the A biotype [18], and this is the first time that this Chlamydia is reported to be infecting other whitefly species with such a high prevalence, sometimes reaching fixation or near fixation, such as in the case of *T. acaciae* populations tested in this study. This high prevalence suggests intimate association between *Fritschea* and *T. acaciae,* and may hint on an important role that this chlamydia is playing in the biology of *T. acaciae. In B. tuberculata,* however, *Fritschea* appeared in only 14% of the tested individuals, and some populations did not harbor it, such is the populations collected from C. Mota and Cardoso (Fig. 2). Since *T. acaciae* and *B. tuberculate* share the same plant host, they are also likely to share common parasitoids. It has been previously shown that *Rickettsia* from *B. tabaci* was acquired by three whitefly parasitoids *Eretmocerus emiratus*, *Eretmocerus eremicus* and *Encarsia pergandiella*, however was not vertically transferred to offspring of these parasotoid species [48]. This study demonstrated the potential routes and barriers to horizontal transmission of symbionts across trophic levels, and suggests that the common symbionts between *T. acaciae* and *B. tuberculate* observed in our study might be also horizontally transferred via parasitization by common whitefly parasitoids, as the ones investigated in this study [48]. Similarly, *Wolbachia* has been shown to be transmitted to the parasitic wasp *Leptopilina boulardi* from its infected host *Drosophila simulans*, and subsequently undergo diminishing vertical transmission in this novel host species [49].

*Arsenophonus* was the only endosymbiont that infected *B. tuberculata* populations but did not appear in *T. acaciae* (Fig. 2). This bacterium was fixed in some populations such as in Assis, close to fixation such is in C. Mota and Cardoso, and appeared only in one individual such as in the case of the populations collected from Votup (Fig. 2). *Arsenophonus* have been implicated in reproductive manipulations in a wide range of insect species by inducing the male-killing phenotype [50], and might be acting like such in *B. tuberculata.* This bacterium was localizaed inside the bacteriosome cells by FISH analysis (Fig. 3), suggesting that it is strictly vertically transmitted and this might be the cause for its absence from *T. acaciae* that colonize the same host plants.

In addition to *Arsenophonus* which was localized to bacteriocyte cells, the other endosymbionts detected in this study were localized in representative specimens. *Hamiltonella* and *Fritschea,* in addition to *Arsenophonus,* were strictly localized inside bacteriocye cells (Fig. 3) in MEAM1, NW2, *B. tuberculata* and *T. acaciae*. *Cardinium* and *Wolbachia* were observed inside and outside bacteriocyte cells (Fig. 3), but appeared in very low concentrations. Only *Rickettsia* strictly appeared outside the bacteriome and was never observed inside (Fig. 3). These localization patterns appeared in all developmental stages: eggs, nymphs and adults. Endosymbionts that are strictly localized to the bacteriocytes are vertically transmitted and thus they may contribute to their host’s fitness [54], similar to effects induced by symbionts localized outside bacteriocytes such as *Wolbachia* and *Rickettsia*. However, those endosymbionts are less likely to manipulate their host’s reproduction since this requires invading reproductive organs outside the bacteriome. *Wolbachia* and *Cardinium* showed dual localization patterns. They can be found in many insect orders at various different frequencies, and were indeed associated with reproductive disorders [51], [53]. Additionally *Wolbachia* is an extensively studied symbiont that has been shown to induce many effects on its arthropod hosts, including effects on mosquito species hosts, an effect that has been recently employed in controlling the spread of the Dengue virus [55], [56]. The low concentration of *Wolbachia* and its dual localization pattern in *B. tuberculata* and *T. acaciae* suggests that it may play a role in reproductive manipulation; however, other effects cannot be ruled out and more research is required to confirm this assumption.

In contrast to the rest of the detected endosymbionts in this study, only *Rickettsia* showed a scattered localization pattern and was observed outside the bacteriome infecting tissues in the abdomen, thorax and head as was recently shown [57]. *Rickettsia* was shown to manipulate host reproduction in many arthropods [50], [52], and this fits well with its localization pattern in *B. tabaci.* Previously, *Rickettsia* has been shown to exhibit two different localization phenotypes: scattered throughout the body and confined to the bacteriome [33]. These two phenotypes were never observed together in the same individuals; however, one study that tested populations from Croatia showed that those two phenotypes were observed in the same individual [27]. Our FISH results showed the presence of only the scattered phenotype in MEAM1 populations where *Rickettsia* was detected (Fig. 2). This phenotype is different from the obligatory *Rickettsia* in booklice, in which it was found to appear with both phenotypes in the same individual [58]. A recent study has demonstrated that *Rickettsia* is transovarially transmitted to *B. tabaci* subsequent generation by invading developing oocytes and follicular cells, thus being included in individuals that develop from those oocytes [57]. *Rickettsia* has been shown to reach high prevalence and near fixation in natural populations as was recently shown in Arizona [59]. The later study has further shown that *Rickettsia-*infected *B. tabaci* females exhibit high fitness benefits such as increased fecundity, a greater rate of survival, and host reproduction manipulation via the production of a higher proportion of daughters [59]. A recent study has shown that *Rickettsia* is horizontally transmitted between individuals through the host plant [39]. Additional studies have shown that the presence of *Rickettsia* in *B. tabaci* populations influenced the whitefly’s response to heat stress by benefitting its host under high temperatures [60], and has also shown to increase the whitefly’s susceptibility to chemical insecticides [61]. Studies on *Rickettsia* from other invertebrates have revealed diverse effects of the bacteria on their hosts. For example, in the pea aphid *Acyrthosiphon pisum*, *Rickettsia-*infected individuals showed lower fresh body weight, reduced fecundity and significantly suppressed densities of *Buchnera,* suggesting a negative effect of *Rickettsia* [62], [63]. Reproductive manipulation and *Rickettsia*-associated parthenogenesis have been shown in the endoparasitoid *Neochrysocharis formosa* [64], and in the parasitoid wasp *Pnigalio soemius* [65], as has *Rickettsia*-associated male-killing in beetles [66], [67], and involvement in oogenesis of booklice [68]. Although none of the later phenomena associated with *Rickettsia* have been investigated in this study, it will be interesting to investigate such interactions of *Rickettsia* with whitefly populations in Brazil.

## Materials and Methods

### Collection of whiteflies

Most of the whitefly samples tested in this study were collected from the state of São Paulo and some samples were collected from the states of Bahia, Minas Gerais and Paraná in eastern Brazil during the years 2010 to 2012. No specific permissions were required for collecting all the samples in this study in all locations presented in Table 1, and this study did not involve endangered or protected species. The insects were collected as adults using a hand-held aspirator and immediately preserved in 100% ethanol or fixative solution (See below) until further processing for endosymbiont or biotype genotyping, or for FISH analysis. Table 1 summarizes the whitefly populations collected and analyzed in this study.

### DNA Extraction and PCR for B and Q biotype identification

DNA was extracted from individual whiteflies as previously described [28]. Four to ten individual adults per population were analyzed for initial species identification using the Bem23 primer pair: Bem23F (5′-CGGAGCTTGCGCCTTAGTC-3′) and Bem23R (5′-CGGCTTTATCATAGCTCTCGT-3′) [30] that distinguish the biotypes B based and Q based on PCR fragment size.

Whitefly species identity confirmation for MEAM1, NW2, *B. tuberculata* and *T. acaciae* was performed by PCR and sequencing using the primer pair C1-J-2195 (5′-TTGATTTTTTGGTCATCCAGAAGT-3′) and L2-N-3014 (5′-TCCAATGCACTAATCTGCCATATTA-3′) that amplify a fragment from the mitochondrial cytochrome oxidase I gene (mtCOI) [31]. For *T. vaporariorum* the primer pair Tvap-F (5′-GGCATTATTTCTCATCTTATTAGTGCT-3′) and Tvap-R (5′-GTGAYTAAGRGMTGGYTTATT-3′) that amplify mtCOI from this species [32] were used. The sequences obtained were verified using BLAST tool in NCBI. Obtained sequences from the different whitefly species were deposited in GenBank and assigned the accession numbers KM368235, KM368236, KM368246–KM368249 and KM368251–KM368255.

### Bacterial endosymbiont

The same DNA originated from each individual was used for the screening of all six secondary endosymbionts *Hamiltonella*, *Rickettsia*, *Wolbachia*, *Arsenophonus*, *Cardinium* and *Fritschea* using genus-specific primers (Table 2) targeting the 16 S or 23 S rDNA genes. PCR cycling was performed as previously described [27], [28]. Confirmation of the presence of the endosymbiont was performed by sequencing the amplified sequences from representative individuals. Obtained sequences from symionts in the different whitefly species were deposited in GenBank and assigned the accession numbers KM368237–KM368245 and KM368250.

### Localization of endosymbionts using Fluorescence in situ Hybridization (FISH)

For endosymbiont localization in adult whiteflies, FISH analysis was used. Generally the methods used in this protocol were previously described [27], [28], [33]. After collecting the insect samples detailed in Table 1, they were immediately transferred to Carnoy’s fixative (Chloroform:Ethanol:Acetic acid = 6∶3∶1) for overnight fixation. The samples were then bleached with 6% H_2_O_2_ in ethanol for 2 hours and hybridized overnight in hybridization buffer (20 mM Tris-HCl [pH 8.0], 0.9 M NaCl, 0.01% SDS, 30% Formamide) containing 10 pmol/ml of fluorescent probes specifically targeting the different symbionts as detailed in Table 3. The specimens were then mounted whole and viewed under an Olympus IX81 Fluoview 500 confocal microscope (Olympus, Tokyo, Japan). At least twenty specimens were analyzed for each endosymbiont in each developmental stage.

**Table 3:** Probes used for endosymbiont localization in whiteflies.

Probe name	Endosymiont	Sequence (5′ 3′)	Reference
BTP1-Cy3	*Portiera*	TGTCAGTGTCAGCCCAGAAG	[20]
Rb1-Cy5	*Rickettsia*	TCCACGTCGCCGTCTTGC	[20]
BTH-Cy5	*Hamiltonella*	CCAGATTCCCAGACTTTACTCA	[13]
Card-Cy5	*Cardinium*	TATCAATTGCAGTTCTAGCG	[71]
Ars2-Cy5	*Arsenophonus*	TCATGACCACAACCTCCAAA	[13]
W2-Cy5	*Wolbachia*	CTTCTGTGAGTACCGTCATTATC	[34]
Frit-Cy5	*Fritschea*	GTCGGGGTTGAGTCCAACTA	This study
